# A Secretory Protein of Necrotrophic Fungus *Sclerotinia sclerotiorum* That Suppresses Host Resistance

**DOI:** 10.1371/journal.pone.0053901

**Published:** 2013-01-14

**Authors:** Wenjun Zhu, Wei Wei, Yanping Fu, Jiasen Cheng, Jiatao Xie, Guoqing Li, Xianhong Yi, Zhensheng Kang, Martin B. Dickman, Daohong Jiang

**Affiliations:** 1 State Key Laboratory of Agricultural Microbiology, Huazhong Agricultural University, Wuhan, Hubei Province, People’s Republic of China; 2 The Provincial Key Lab of Plant Pathology of Hubei Province, College of Plant Science and Technology, Huazhong Agricultural University, Wuhan, Hubei Province, People’s Republic of China; 3 State Key Laboratory of Crop Stress Biology for Arid Areas, Northwest A&F University, Yangling, Shaanxi, People’s Republic of China; 4 Institute for Plant Genomics and Biotechnology, Department of Plant Pathology and Microbiology, Texas A&M University, College Station, Texas, United States of America; Nanjing Agricultural University, China

## Abstract

*SSITL* (*SS1G_14133*) of *Sclerotinia sclerotiorum* encodes a protein with 302 amino acid residues including a signal peptide, its secretion property was confirmed with immunolocalization and immunofluorescence techniques. SSITL was classified in the integrin alpha N-terminal domain superfamily, and its 3D structure is similar to those of human integrin α4-subunit and a fungal integrin-like protein. When *S. sclerotiorum* was inoculated to its host, high expression of *SSITL* was detected during the initial stages of infection (1.5–3.0 hpi). Targeted silencing of *SSITL* resulted in a significant reduction in virulence; on the other hand, inoculation of *SSITL* silenced transformant A10 initiated strong and rapid defense response in Arabidopsis, the highest expressions of defense genes *PDF1.2* and *PR-1* appeared at 3 hpi which was 9 hr earlier than that time when plants were inoculated with the wild-type strain of *S. sclerotiorum*. Systemic resistance induced by A10 was detected by analysis of the expression of *PDF1.2* and *PR-1*, and confirmed following inoculation with *Botrytis cinerea*. A10 induced much larger lesions on Arabidopsis mutant *ein2* and *jar1*, and slightly larger lesions on mutant *pad4* and *NahG* in comparison with the wild-type plants. Furthermore, both transient and constitutive expression of *SSITL* in Arabidopsis suppressed the expression of *PDF1.2* and led to be more susceptible to A10 and the wild-type strain of *S. sclerotiorum* and *B. cinerea*. Our results suggested that SSITL is an effector possibly and plays significant role in the suppression of jasmonic/ethylene (JA/ET) signal pathway mediated resistance at the early stage of infection.

## Introduction

*Sclerotinia sclerotiorum* (Lib.) de Bary is an economically significant and destructive necrotrophic fungal pathogen with the capability of infecting more than 450 species and subspecies of plants worldwide [1], [2]. Sclerotinia diseases lead to serious losses each year in both vegetable crops and plant oil crops, including rapeseed, soybean and sunflower. At the latter stages of infection, *S. sclerotiorum* produces dormant melanized sclerotia in soil and diseased stubbles during summer or/and winter. This durable differentiated structure can persist and maintain viability in the soil for many years. Host differentials of this pathogen have not been observed and strains isolated from one host usually have similar virulence to other hosts. Sclerotinia diseases have proven difficult to control as breeding efforts have not met with success, resistance is complex, useful cultivars are not available and management practices and chemical spray regimes are ineffective. Thus the control of Sclerotinia diseases has raised much concern.

Plant pathogen life styles have been divided into biotrophic, hemibiotrophic and necrotrophic pathogens. Biotrophic pathogens must derive nutrients from living host cells and tissues, hemibiotrophic pathogens absorb nutrients from living cells at the early biotrophic stages of infection and transition into a necrotroph killing host cells for nutrient acquisition. Necrotrophic pathogens kill host cells and/or feed on dead tissue. Often, necrotrophic pathogens secrete toxins (including non-host-selective toxins and host-selective toxins), plant cell wall degrading enzymes, and proteinases to facilitate host cell death.The interaction between necrotrophic fungal pathogens and hosts is clearly more complex than originally thought. Rather than overwhelming the plant host with its battery of enzymes and toxins, evidence is emerging that these fungi are more subtle in their pathogenic tactics. Oirdi *et al* [3] reported that *Botrytis cinerea* manipulates the antagonistic effects between immune pathways to promote disease development in tomato, where *B. cinerea* produces a EPS b-(1,3)(1,6)-D-glucan to activate the SA signal pathway. The SA signal pathway inhibits JA signaling through NPR-1, resulting in enhanced host susceptibility. When challenging Arabidopsis, *B. cinerea* induces the expression of autophagy genes. Arabidopsis mutants defective in autophagy exhibit enhanced susceptibility to *B. cinerea* and *Alternaria brassicicola* [4]. On the other hand, over-expression of a *B. cinerea* anti-apoptotic gene *BcBIR1* in *B. cinerea* enhances virulence [5]. Plants also activate defense systems against necrotrophic fungal pathogens. Many P/DAMPs, such as fragments of chitin from fungal cell walls and the plant cell wall–derived oligogalacturonides (OGs) can be recognized by membrane localized receptor-like kinases (RLKs), such as the putative chitin receptor LysM/CERK1 [6], [7], peptide receptors [8], [9], and the OG receptor WALL-ASSOCIATED KINASE 1 (WAK1) [10], and then activate the immune responses to necrotrophic pathogens. Although plant-produced ROS are important for resistance to biotrophs and hemibiotrophs, the role of ROS in resistance to *S. sclerotiorum* appears to be more complex, with a resistance role during early infection, but once infection is established, promoting disease during later infection [11]. Plant hormones also modulate innate immunity to necrotrophs. In general, SA signaling pathway is activated against biotrophic pathogens, whereas jasmonic/ethylene (JA/ET) signal pathway is effective against necrotrophs [12]–[14]. However, many previous studies have shown that SA-mediated defense is also involved in the resistance to necrotrophic [15], [16]. Cross-talk between SA and JA/ET signaling pathways has been shown to optimize the defense response against the attacker [17]–[19]. Together, evidence suggests that strong interaction between necrotrophic pathogens and hosts should exist before killing and being killed.

*S. sclerotiorum* is believed to be a typical necrotrophic fungus, originally viewed as an aggressive pathogen secreting copious amounts of oxalic acid (OA) to rapidly kill host cells and tissues, coupled with a number of cell-wall degrading enzymes to further destroy plant tissues during infection [20]. However, OA is likely to have more important roles, it suppresses host oxygen burst and host resistance [21], [22], and triggers ROS mediated apoptotic-like PCD. Recently, OA was found to create reducing conditions in plant cells ahead of advancing hyphae. It was speculated that reductive conditions dampen the oxidative burst allowing for precious time for fungal establishment prior to plant recognition. Furthermore, in the compatible interactions between *S. sclerotiorum* and its host, host cells maintain viability, and at the same time, suppression of the oxidative burst and callose deposition is observed, in a manner akin to compatible biotrophic pathogens during the early stage of infection [11]. Thus, *S. sclerotiorum* is more accurately characterized as hemi-biotrophic pathogen possibly.

Previously, we reported that certain strains of *S. sclerotiorum* harbored a debilitation associated RNA mycovirus (SsDRV) resulting in a hypovirulent phenotype. Studies designed to identify fungal genes down-regulated by the infection of SsDRV uncovered a gene encoding a protein similar to integrin. This *Sclerotinia sclerotiorum integrin-like* gene (*SSITL*) was significantly suppressed by the RNA virus infection [23]. Integrins belong to a large family of cell surface protein molecules that act as conserved transmembrane cell-adhesion receptors in a variety of vertebrates and invertebrates [24]. They play a critical role in cell structure, cell migration, anchoring cells to the extracellular matrices and carrying signals from the outside to the inside of the cell and vice versa. These mechanical and chemical signals play significant roles in cell cycle, growth, development, differentiation, proliferation and apoptosis [25]–[27]. Integrin-like proteins were also found in plants [28], [29], and were considered to have similar functions as reported in animals. Recently, the non-race specific disease resistance *NDR1* gene in Arabidopsis has been shown to be an integrin-like protein gene [30].

Fungal integrin-like proteins were also identified with immunobiological or pharmacological methods. The first integrin-like protein was identified from yeast *Candida* by immunobiological assay [31]; an integrin-like protein was identified in *Uromyces appendiculatus* with RGD short peptide emendation test [32]. By screening an expression library with two antibodies against leukocyte integrins αX and αM, the gene encoding *integrin-like uso1* was isolated from *Saccharomyces cerevisiae* [33], and gene cloned from *Candida albicans* was another fungal *integrin-like* gene *αint1*, which has a limited similarity to vertebrate integrins and contributes to hyphal growth and adhesion to epithelial cells. The integrin-like protein in *C. albicans* is essential for the virulence in murine model of intravenous infection [34], [35]. Integrin-like proteins in plant fungal pathogens are likely to involve in surface attachment and pre-penetration stage development [36]. However, whether integrin-like proteins contribute to the virulence of plant fungal pathogen is still unknown.

To characterize the function of this gene, we employed both forward and reverse genetic approaches. We report that SSITL, a potential effector is involved in suppressing host resistance at the early stage of infection.

## Materials and Methods

### Fungal Strains, Plants, and Culture Condition

*S. sclerotiorum* virulent strain Ep-1PNA367 was derived from the single-ascospore-isolation progeny of virus-infected hypovirulent strain Ep-1PN, and CanBc-1c-66 was a single-conidium strain of *B. cinerea* isolated from rapeseed [37]. Fungal cultures were grown on potato dextrose agar (PDA) (Difco, Detroit, MI, USA) at 20°C. *S. sclerotiorum* transformants were obtained and purified by up to ten rounds of hyphal tip subculture on PDA amended with 30 µg/mL hygromycin B (EMD Biosciences, USA) to stabilize transformants. *Escherichia coli* strain JM109 was used to propagate all plasmids, while *Agrobacterium tumefaciems* strain EHA105 and GV3101 were used for transformation. *Arabidopsis thaliana* wild-type Columbia-0 and mutant *ein2-1*, *jar1-1*, *NahG* and *pad4-1* were kindly donated by Dr Yangdou Wei in University of Saskatchewan, and were grown in a greenhouse at 20±2°C, under a 12 hr light/dark cycle.

### Bioinformatics Data and Programs Used in this Study

The publicly available genomic sequence database of *S. sclerotiorum* (http://www.broadinstitute.org/annotation/genome/sclerotinia_sclerotiorum/MultiHome.html) was used to characterize gene *SSITL* (*Sclerotinia sclerotiorum integrin-like*). The *TMHMM Server v. 2.0* (http://www.cbs.dtu.dk/services/TMHMM/), the *Signal P 3.0 Serverits* (http://www.cbs.dtu.dk/services/SignalP/) and the *MultiLoc/TargetLoc* (http://abi.inf.uni-tuebingen.de/Services/MultiLoc/) were used to predict the transmembrane domain, signal peptide sequence and subcellular localization of protein SSITL, respectively. *Bioinformatics Toolkit HHblits* (http://toolkit.tuebingen.mpg.de/hhblits), *NCBI* (http://www.ncbi.nlm.nih.gov/) and *UniProt* (http://www.uniprot.org/) were used for Blastp analysis. The *ClustalX* and *MCOFFEE program* (http://tcoffee.vital-it.ch/cgi-bin/Tcoffee/tcoffee_cgi/index.cgi?stage1=1&daction=MCOFFEE::Advanced) were used for amino acid alignments. The *InterProScan Sequence Search* (http://www.ebi.ac.uk/Tools/pfa/iprscan/) and *SUPERFAMILY HMM search* (http://supfam.org/SUPERFAMILY/hmm.html) were used to predict the protein superfamily. The *Radar* (http://www.ebi.ac.uk/Tools/Radar/), *Bioinformatics Toolkit HHpred* (http://toolkit.tuebingen.mpg.de/hhpred) and the *Jnetpred* were used to predict the structure of SSITL. The 3D structural model was established by using the *Phyre2 server* (http://www.sbg.bio.ic.ac.uk/phyre2/html/page.cgi?id=index).

### Construction of *SSITL* Vectors and Transformation of *S. sclerotiorum*

The strategy to construct a *SSITL* RNAi silence vectors was performed as described by Yu *et al* [39]. A 452 bp DNA fragment was PCR amplified from th*e Integrin-like* (SS1G_14133) with a pair of specific primers. At the 5' terminus of the sense primer, two restriction sites (*Bam*HI and *Cla*I) were introduced, and at the 5' terminus of the antisense primer, restriction sites for *Pst*I and *Eco*RV were introduced. The primer sequences are listed in Table 1. The PCR product was co-digested with *Bam*HI and *Pst*I or with *Cla*I and *Eco*RV to generate DNA fragments with two types of cohesive ends. The two fragments were ligated to pCIT to generate a new vector containing a reverse repeat structure that was separated by the 420 bp intron was amplified from a *Gibberella zeae* gene (EAA75655.1). This newly constructed vector was then digested with *Xho*I and *Sac*I to obtain the repeat fragment, and then ligated with the pCH vector also digested by *Xho*I and *Sac*I to generate the *integrin-like* silencing vector pIntSILENCE. The pIntSILENCE vector was transformed into *A. tumefacies* strain EHA105.

**Table 1:** Primers used for vector construction and RT-PCR.

Application of primer pair	Primer’s direction	Primer sequence
*SSITL* silence vector, for amplifying intron from *G. zeae* gene(EAA75655.1)	Sense	5′GCGATATCAGGCAGCGTGAGTTTAC 3′
	Antisense	5′TGCACTGCAGCCTACTCAGACTGGACA 3′
*SSITL* silence vector, for amplifying *SSITL* gene from *S. sclerotiorum*	Sense	5′ CGCGGATCCATCGATAGCGTAATGGATGGTGG 3′
	Antisense	5′ CGTCTGCAGGATATCAAGCAGATGCGACGAAC 3′
*SSITL* prokaryotic expression vector	Sense	5′ CGGGATCCGATCCCAAACCCCCTTGAGAAACG 3
	Antisense	5′ CCCAAGCTTACCACTAGCAACATGTACTTCG 3′
*SSITL* expression vector in host plants	Sense	5′ CGGGATCCATGCTGCTCAAACCACTT 3′
	Antisense	5′ CGAGCTCTCAACCACTAGCAACATGTAC 3′
*A. thaliana GAPDH* gene (At1g13440) used for RT-PCR	Sense	5′ GCAACATACGACGAAATCAAGAA 3′
	Antisense	5′ CGACACGAGAACTGTAACCCC 3′
*N. benthamiana actin* gene (AY179605.1) used for RT-PCR	Sense	5′ GCCGAGCGGGAAATTGTTAGG 3′
	Antisense	5′ CCACTGAGGACAATGTTTCCGTAC 3′

To construct an *E. coli* expression vector, the full-length cDNA of *SSITL* gene without the signal sequence was amplified by PCR with a pair of specific primers. Restriction sites (*Bam*HI and *Hin*dIII) were introduced at the 5' terminus of sense primer and antisense primer, respectively. Primers sequences are listed in Table 1. The pET-22b (+) vector and the PCR products were digested by *Bam*HI and *Hin*dIII, and the cDNA fragment was ligated with pET-22b (+) to generate the expression vector pET22bInt.

To transform *S. sclerotiorum*, protoplasts of strain Ep-1PNA367 were prepared as described by Rollins [38]. Agrobacterium-mediated transformation (ATMT) of *S. sclerotiorum* was performed as described by Yu *et al* [39] with some modifications that for co-cultivation, the *S. sclerotiorum* protoplasts were re-suspended with *A. tumefaciens* at the concentration of 1×10^8^ protoplasts per ml and cultured on a cellophane membrane laid on co-induction medium. Hygromycin was amended into PDA at a final concentration of 30 µg/mL.

### Extraction and Manipulation of Nucleic Acids

To examine the expression pattern of *SSITL* in different growth stages of *S. sclerotiorum*, mycelial agar discs taken from the active colony edge of Ep-1PNA367 were inoculated on the cellophane over PDA at 20°C. The mycelia were collected at 1, 2, 3, 4 and 5 day post incubation (dpi), and then stored at −80°C for total RNA extraction. To explore *SS1TL* gene expression during fungal interaction with Arabidopsis, 4 g fresh Ep-1PNA367 mycelia was ground into fragments using a sterile mortar and pestle. The hyphal fragments were cultured in 100 ml minimal medium broth in a 250 ml flask at 20°C, at 150 rpm and fragments were collected by centrifugation and washed with ddH_2_O twice before being re-suspended in 20 ml ddH_2_O. The hyphal fragments suspension was sprayed onto the leaves of *A. thaliana* Columbia-0 (6–8 weeks-old). The inoculated leaves and the hyphae growing in plates as control, were harvested at 1.5, 3, 4.5, 6, 7.5, 9, 10.5 and 12 hours post inoculation (hpi), respectively, and then stored at −80°C. To compare transcript accumulation of *SSITL* between silenced transformants and the wild-type strain (Ep-1PNA367), the active mycelial agar discs of silenced transformants and Ep-1PNA367 were inoculated to the cellophane of PDA at 20°C for 3 days (transcript levels reached the peak in Ep-1PNA367 at 3 dpi) and mycelium was then collected and stored at −80°C for total RNA extraction.

The total RNA samples of fungal strains and plants were isolated with TriZOL reagent (Invitrogen, USA) according to the manufacturer’s protocols. Northern hybridization analysis was performed as previously described by Li *et al* [23]. The cDNA of *SSITL* gene was labeled with ^[32^P^]^ dCTP using a radiolabeling kit (TaKaRa, Dalian) probes. The total RNA samples were treated with DNase I (TaKaRa, Dalian), and used to generate the first strand cDNA with RevertAid™ First Strand cDNA Synthesis Kit (MBI Fermentas, Lithuania). Gene expression was analyzed by Real-Time (quantitative) RT-PCR using a Bio-Rad CFX96 Real Time System (America) and Quantitect SYBR Green PCR master mix (Bio-Rad, USA), according to the manufacturer’s instructions. Primers were designed across or flanking an intron (See Table 2 for primers and PCR conditions). The expression of *S. sclerotiorum β-tubulin* gene (SS1G_04652) [40] and *A. thaliana GAPDH* (AT1G13440) were used to normalize the RNA sample for each Real-time RT-PCR. For each gene, Real-time RT-PCR assays were repeated at least twice, with each repetition having three replicates.

**Table 2:** Primers and conditions used for Real-time RT-PCR amplification.

Primer name	Sequence (5′ to 3′)	Target organisms	Target gene	Annealing temperature (°C)	PCR product size (bp)	Acquiring temperature (°C) in Real-time RT- PCR
*At.PDF1.2* F	TCTTCGCTGCTCTTGTTCTCTT	*Arabidopsis thaliana* genes	*At.PDF1.2* (AT5G44420.1)	55	150	72
*At.PDF1.2* R	TGGCTCCTTCAAGGTTAATGC					
*At.PR-1* F	CTACGCAGAACAACTAAGAGGC		*At.PR-1* (AT2G14610)	55	150	72
*At.PR-1*R	TTCGCAGCGTAGTTGTAGTTAG					
*At.GAPDH* F	GCAACATACGACGAAATCAAGAA		*At.GAPDH* (AT1G13440)	55	217	72
*At.GAPDH* R	CGACACGAGAACTGTAACCCC					
*Ss. SSITL* F	AAGAGCGTAATGGATGGTGG	*Sclerotinia sclerotiorum* genes	*SS1G_14133*	56	167	72
*Ss. SSITL* R	AGCAAATGTGGTGCCGACT					
*Ss. β-tubulin* F	TTGGATTTGCTCCTTTGACCAG		*β-tubulin* (SS1G_04652)	56	104	72
*Ss.β-tubulin* R	AGCGGCCATCATGTTCTTAGG					

Primer pairs for PCR amplifications, RT-PCR amplification and Real-Time PCR detections were listed in Table 1 and Table 2.

### Characterization of *SSITL* Silenced Transformants

To assay growth rates, the silenced transformant and the virulent strain Ep-1PNA367 were cultivated on PDA at 20°C for 3 days. The mycelial agar discs were taken from the active colony edge and inoculated on the center of the PDA *petri* dish at 20°C before hyphal growth was examined. After growth on PDA at 20°C for 48 hr, the tip hyphal morphology of the silenced transformants and the wild-type strain Ep-1PNA367 were observed under a light microscope. The colony morphology and sclerotia distribution of these strains were examined after being grown on PDA plate for 30 days at 20°C. The mycelial agar discs of these strains were inoculated to steam-sterilized carrot in triangular flasks at 20°C for 30 days to culture sclerotia for analysis.

To evaluate virulence, mycelial agar discs (diameter 6 mm) were inoculated to the detached *Brassica napus* leaves at 20°C for 72 hr, and lesions induced by transformants were measured.

### Subcellular Localization of SSITL in *S. sclerotiorum* and in Host Cells

To prepare the antiserum of SSITL, pET22bInt was transformed to *E. coli* strain Rosetta (DE3). Expression of the target protein (*SSIT*) in *E. coli* was performed according to Novagen. pET System Manual. 11^th^ Edition. The purification and expression of SSITL antiserum were performed according to the methods of Xu *et al* [41].

To study cellular localization of the SSITL protein in hyphal cell, A10 and the wild-type strain were grown on PDA for 72 hr and then the mycelial agar discs from the active colony edge were collected. The distribution of SSITL during plant infection was determined by inoculating mycelial agar discs to the leaves of 6–8 week-old plants of Col-0 for 12 hr at 20°C. Diseased leaves were collected. Sample preparation, immunogold labeling were performed according to the methods described by Kang *et al* [42].

Immunofluorescence with minor modifications was also used to confirm the secretion of SSITL [43]. Transgenic *S. sclerotiorum* strains carrying a SSITL: Flag tag fusion protein was expressed under the modulation of *PtrpC*. The expression of the fusion gene was determined by RT-PCR, and confirmed by Western blot analysis. One transformant was chosen as a representative and inoculated to onion bulb epidermal tissue for 12–24 hr at 20°C. The inoculated epidermis was rolled with forceps, washed with PBS buffer for three times and fixed with 2 ml 4% paraformaldehyde for 15 min. The fixed epidermis was permeabilized in 0.1% Triton X-100 or 0.1% NP-40 buffer for 3 min, blocked for 15 min in PBS containing 1% BSA and 0.09% sodium azide, and then incubated with the primary anti Flag-tag mouse monoclonal antibody (1∶50 dilution in PBS containing 1%BSA) (CWBIO, China) at room temperature for 2 hr. The epidermis was washed for three times with PBS buffer and incubated with the secondary antibody (Goat anti-mouse-Rhodamine Red-X, 1∶75 dilution in PBS containing 1% BSA) (CWBIO, China) for 1 hr at room temperature. After three times of washing with PBS buffer, the immunofluorescence reaction was observed under a Nikon Eclipse 80i fluorescent microscope (Nikon, Japan). Ep-1PNA367 was used as control. The excitation wavelength and emission wavelength used here were 510–560 nm and 575–590 nm, respectively.

### Influence of SSITL to Local and Systemic Resistance

To explore SSITL with respect to pathogenicity, local resistance and systemic resistance assays were conducted. To probe local resistance affected by the *SSITL* silenced transformant, mycelial agar discs (diameter 4 mm) were taken from the active colony edge and inoculated on leaves of 6–8 week-old *A. thaliana* Columbia-0 and mutant *ein2*, *jar1*, *NahG* and *pad4* at 20°C. The wild-type strain Ep-1PNA367 was inoculated on the leaves of Arabidopsis as control. The inoculated leaves of Arabidopsis were harvested at 3, 6, 9, and 12 hpi, respectively, and then stored at −80°C for RNA extraction and Real-Time RT-PCR analysis. A portion of inoculated plants were further incubated for 36 hr at the same condition to allow lesion development, and then lesions were photographed and their diameters were measured.

To probe the systemic defense responses induced by A10, the lower leaves of 6–8 weeks old *A. thaliana* Columbia-0 and mutant *ein2, jar1, NahG* and *pad4* were inoculated with the mycelial agar of A10 for 48 hr at 20°C. Water agar and Ep-1PNA367 were inoculated to Arabidopsis as controls. Inoculated leaves were cut with a sterilized scissors at 48 hpi, and upper leaves were inoculated with *B. cinerea* mycelial agar discs. The *B. cinerea*-inoculated leaves were collected at 0, 1, 2 and 3 days post-inoculation and stored at −80°C for RNA extraction and Real-Time RT-PCR analysis. The lesions induced by *B. cinerea* on the leaves of Arabidopsis were measured and photographed at 72 hpi.

The expression of two defense marker genes, *PDF1.2* (AT5G44420.1) for JA/ET signal pathway mediated resistance and *PR-1* (AT2G14610) for SA signal pathway mediated resistance, were examined with Real-Time RT-PCR assay as described above.

### Transient and Constitutive Expression of *SSITL* in Host Plants

To construct an expression vector, full-length *SSITL* cDNA including the signal sequence, was amplified by PCR with a pair of specific primers. The restriction sites of *Bam*HI and *Sac*I were introduced at the 5' terminus of sense and antisense primers, respectively. The pBI121 vector and the PCR products were digested by *Bam*HI and *Sac*I, and the cDNA fragment was ligated into pBI121 to generate the expression vector pBI121-Int. pBI121-Int was transformed to *A. tumefaciems* strain GV3101. For *SSITL* transient assays, Agrobacterium-mediated transient expression was performed using leaf infiltration as described by Krasileva *et al* [44]. For the *SSITL* constitutive expression, the Agrobacterium-mediated transformation of *A. thaliana* was performed using the floral dip method as described by Zhang *et al* [45].

To determine whether SSITL affects on local resistance, tobacco leaves were infiltrated with Agrobacterium GV3101 strain carrying the *SSITL* expression vector, and then the *SSITL* transiently expressed leaves of tobacco were inoculated with A10 and Ep-1PNA367, respectively. The plants were inoculated for 48 hr at 20°C, and then the sizes of lesions induced by A10 and Ep-1PNA367 were measured. Arabidopsis leaves also were infiltrated and inoculated with A10 or Ep-1PNA367 for 48 hr or 24 hr, respectively, and then the lesions diameters were measured. Leaves infiltrated with GV3101 carrying empty vector were served as control. Expression levels of defense genes *PDF1.2* and *PR-1* in inoculated leaves were monitored as described above. In terms of systemic resistance, the *SSITL* transiently expressed leaves of *A. thaliana* were inoculated with A10 for 48 hr. The inoculated leaves were cut and the upper leaves were inoculated with *B. cinerea* mycelial agar discs for 72 hr at 20°C, and then the lesion diameters were measured. Expression levels of defense genes *PDF1.2* and *PR-1* in upper leaves were monitored after the *SSITL* transiently expressed leaves were inoculated with A10 for 48 hr. Leaves infiltrated with GV3101 carrying empty vector were served as control.

In the constitutive expression of *SSITL*, the *SSITL* transgenic *A. thaliana* was inoculated with A10 for 72 hr or with Ep-1PNA367 for 24 hr at 20°C, respectively, and then the lesions diameters were measured. The expression assays of resistance genes *PDF1.2* and *PR-1* in *SSITL* transgenic lines of *A. thaliana* were performed. Alternatively, the *SSITL* transgenic lines of *A. thaliana* were inoculated with *B. cinerea* for 24 hr at 20°C before the lesions diameters were measured.

## Results

### SS1G_14133 has a Secretion Property and is Similar to Integrin-like Protein

**Figure 1:**
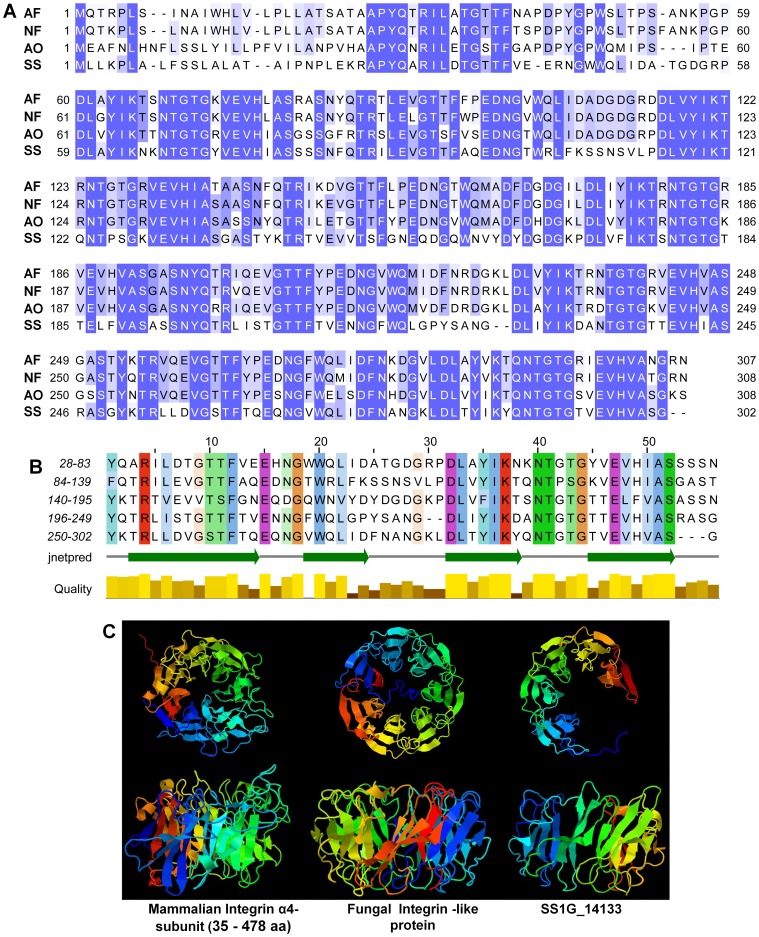
Characterization of the *S. sclerotiorum SS1G_14133* gene. (A) Alignment of the amino acid sequences of SS1G_14133 protein of *S. sclerotiorum* and other organisms using MCOFFEE and ClustalX program. AF: *Aspergillus fumigates* (XP_750162.2); NF: *Neosartorya fischeri* (XP_001265249.1); AO: *A. oryzae* (EIT81778.1). (B) Alignment of the repeat peptides sequences and prediction of secondary structure of SS1G_14133 protein. These alignments were obtained using the MCOFFEE and ClustalX program and the default color scheme for ClustalW alignment in the Jalview program was used. The secondary structure prediction was completed with Jnetpred program–beta strands as green arrows. Quality (yellow) is the quality level for the multiple alignments. (C) The comparison of the 3D structural models of SS1G_14133 protein, Integrin α4-subunit (from 35 aa to 478 aa) (*UniProt* Id: P13612) [46] and *Psathyrella velutina* Integrin-like protein *(UniProt* Id: Q309D1) [47]. The images were obtained from the top (upper) and side (lower) of these proteins. The 3D structural models were generated with Phyre2 program.

The *S. sclerotiorum SS1G_14133* gene is a single copy gene, consisting of two exons and one intron and encoding 302 amino acid residues and the initial N terminus 17 amino acids encode signal peptide. No transmembrane helices of this protein were predicted and the entire amino acid sequence/protein is outside the cell, thus, the protein coded by *SS1G_14133* is a secretory protein possibly. BLAST searches for homologous sequences resulted in significant similarity with sequences from *B. fuckeliana* (CCD48260.1, E-value: 6.3E-152), *Aspergillus fumigatus* (XP_750162.2, E-value: 2E-105), *Neosartorya fischeri* (XP_001265249.1, E-value: 3E-102), *A. oryzae* (EIT81778.1, E-value: 5E-105) that match to the FG-GAP repeat domain-containing proteins frequently found in the N terminus of integrin alpha chains [46]. Sequence alignment of these homologs revealed significant conservation in length and amino acid composition (Figure 1A), except the homolog from *B. fuckeliana*, which has 584 amino acid residues. Internal sequence repeats and secondary structure prediction analysis show that SS1G_14133 protein contains five highly conserved repeats; each repeat consists of four β strands (Figure 1B). Furthermore, both the search results using *Superfamily HMM Sequence Search* and *InterProScan Sequence Search* indicated that this protein is classified in the integrin alpha N-terminal domain superfamily (each E-value is 1.01E-10 and 6.7E-6 respectively). The *Bioinformatics Toolkit HHpred* analysis result indicates that the function and structure of SS1G_14133 is similar to human (*Homo sapiens*) integrin α subunits (*UniProt* Id: P06756, E-value: 1.6E-16). The 3D structural model predicted by *Phyre2* showed that SS1G_14133 adopts a regular five-bladed β-propeller domain with each blade consisting of four β strands (Figure 1C), and shares a strong structural similarity with the integrin α4-subunit (*UniProt* Id: P13612, E-value: 1.2E-21, Confidence: 99.90%, Score: 114.97) [46] and *Psathyrella velutina* integrin-like fungal protein (*UniProt* Id: Q309D1, E-value: 1.3E-30, Confidence: 100.00%, Score: 155.33) [47]. However, both of these two proteins contain a seven-bladed β-propeller domain.

In summary, the protein coded by *SS1G_14133* resembles integrin-like proteins, thus we named this gene “*SSITL*” derived from *Sclerotinia sclerotiorum integrin-like* gene.

### *SSITL* Expresses Highly at the Early Stages of Infection and Sclerotial Development

**Figure 2:**
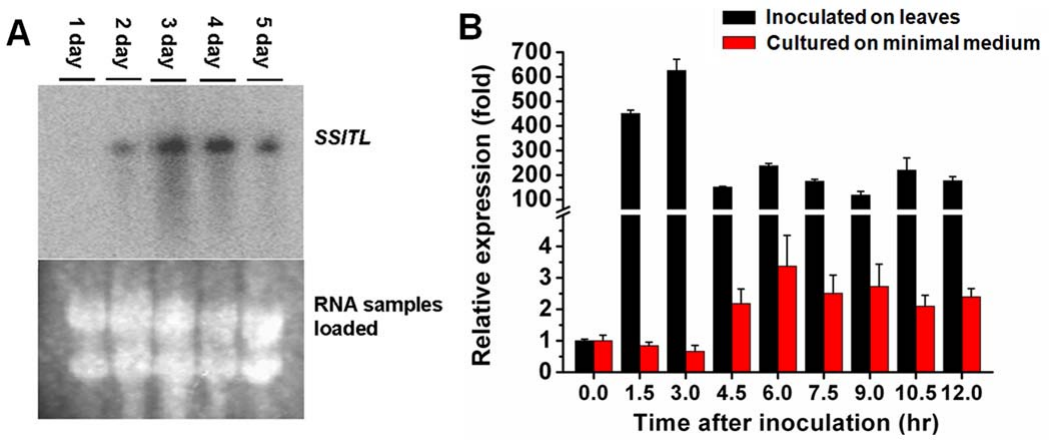
Gene expression analysis of *SSITL gene in the wild-type strain Ep-1PNA367 of* *S. sclerotiorum*. (A) The Northern blot analysis shows gene expression levels of *SSITL* grown on PDA from 24 hr to 120 hr, respectively. The rRNA levels on the nylon membrane transferred from the ethidium bromide (EtBr)-staining of the gel (lower) were used as sample loading marker. (B) The relative transcript accumulation patterns of *SSITL* gene detected with Real-time RT-PCR amplification after contacting with Arabidopsis plants (dark columns) or growing on minimal medium (red columns) for 0–12 hr. The relative levels of transcript were calculated by the comparative Ct method. The *SSITL* gene expression of *S. sclerotiorum* inoculated on plants or in plate at 0 hr was set as level one. The levels of *β-tubulin* transcript were used to normalize different samples. Bars represent means and standard deviations (three replications).

Northern blot was used to examine the expression pattern of *SSITL* at several stages of mycelial growth on PDA. Results showed that expression of *SSITL* was detected on the second day but not on the first day; transcript accumulation peaked on the third day and then decreased slightly on the 4^th^ day and the 5^th^ day (Figure 2A). When grown on PDA for 3–4 days, *S. sclerotiorum* initiated sclerotial development; thus suggested that SSITL is involved in sclerotial development possibly.

Real-Time RT-PCR analysis was used to determine expression patterns of *SSITL* during fungal interaction with host plants. Results indicated that when actively growing hyphal fragments of *S. sclerotiorum* were inoculated on leaves of *A. thaliana* (Col-0), the transcript levels of *SSITL* rapidly increased (600 fold) peaking at 3 hpi, and then decreased, but expression was still about 120–250 fold higher than at 0 hpi for and remained so for a further 9 hr (Figure 2B). However, when the same mycelial fragments were inoculated on minimal medium, the expression of *SSITL* did not vary to any great extent, with the highest expression being observed at 6 hpi and the relative expression being about 3.4 fold higher than at 0 hpi (Figure 2B). Thus the expression of *SSITL* was strongly induced by its interaction with host. And also, when inoculated with the wild-type strain Ep-1PNA367, lesions on the *A. thaliana* leaves could be observed at approximately 6 hpi, correlating with the expression pattern of *SSITL* during infection, suggested that this gene may play significant roles at the early stages of infection.

### *SSITL* Silenced Transformants Show Abnormal Phenotype

**Figure 3:**
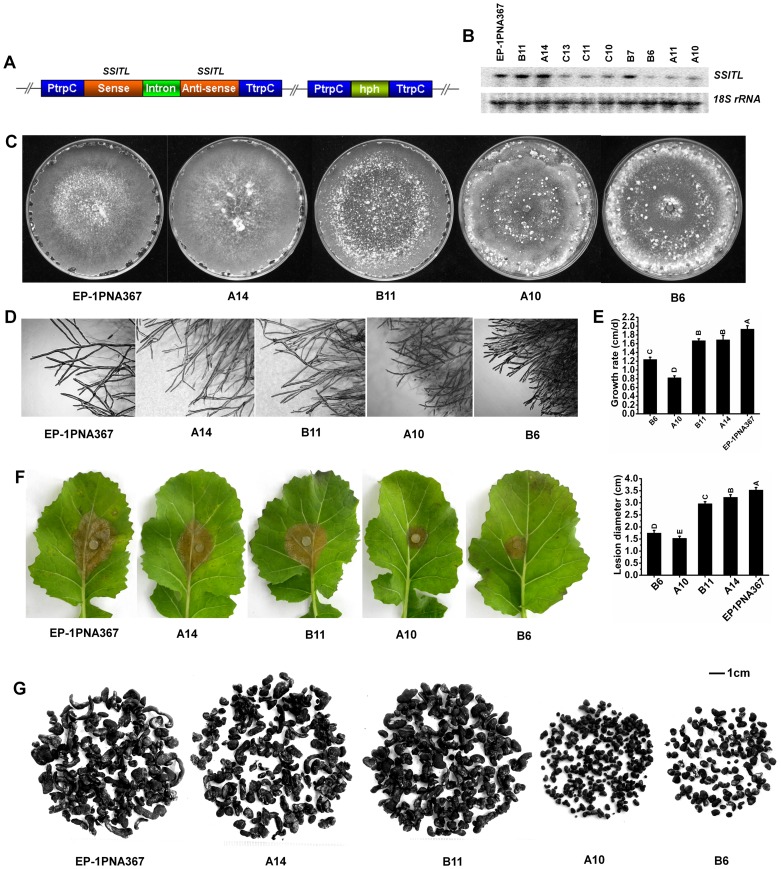
*SSITL* silenced transformants showing abnormal phenotypes. (A) The construction of *SSITL* silenced vector. (B) Northern blots analysis of the *SSITL* gene transcript accumulation in *SSITL* silenced transformants. Expression of *SSITL* in the wild-type Ep-1PNA367 served as control. Hyphae mass from 3-day-old colonies on PDA was collected for gene expression analysis. RNA samples were monitored by Northern hybridization analysis of 18S rRNA on the nylon membrane. (C) Abnormal colony morphology produced by *SSLTL* silenced transformants. Colonies were grown on PDA for 30 days at 20°C. (D) Excessive branching of hyphal tips of *SSITL* silenced transformants. (E) Hyphal growth rates of *SSITL* silenced transformants. Growth rates were examined on PDA at 20°C. Different letters in the graph indicate statistical differences, *P* = 0.01. (F) Virulence decreases in *SSITL* silenced transformants. Virulence was evaluated on detached leaves of rapeseed (*Brassica napus*) measured by the lesions diameter at 20°C for 72 h. Different letters in the graph indicate statistical differences, *P* = 0.01. (G) Sclerotial sizes of *SSITL* silenced transformants. Sclerotia were produced on the autoclaved carrot rods in 250 ml flasks at 20°C for 30 days.

To study the functions of *SSITL*, this gene was silenced with the RNAi technique. A gene silencing vector (Figure 3A) was used to transform the wild-type strain Ep-1PNA367. Northern blot analysis was used to examine the transcript accumulation of *SSITL* in each transformants. The *SSITL* expressions in six transformants were found to be dramatically reduced after up to ten rounds of hyphal tip purification (Figure 3B). Colony morphology, growth rate, virulence and the tip hyphae morphology of *SSITL* silenced transformants were further studied.

*SSITL* affects hyphal growth and colony morphology. *SSITL* silenced transformants sectored on PDA with abnormal colony morphology (Figure 3C). Microscopic observations of hyphal tips from growing colonies of *SSITL* silenced transformants showed more excessive and shorter tip branching, denser hyphae (Figure 3D), and reduced in growth rate (Figure 3E).

As suggested, *SSITL* appears to be important for sclerotial development. When grown on PDA, the *SSITL* silenced transformants produced abundant irregular sclerotia. The sclerotia varied in size from each other in the same plate, but were smaller than those produced by the wild-type strain. In contrast, Ep-1PNA367 produced sclerotia at or near the outer edge of the plate (Figure 3C). Many sclerotial initials formed but failed to fully develop (Figure 3C).

To confirm the effect of *SSITL* on sclerotial development, *SSITL* silenced transformants were cultured on autoclaved carrot in 250 ml flasks at 20°C for 30 days and sclerotia were collected. Sclerotia of *SSITL* silenced transformants also decreased remarkably in size and most of which were spherical in shape, while the sclerotia of the wild-type strain were irregular in shape (Figure 3G). The weight per hundred sclerotia of *SSITL* silenced transformants A10 and B6 were 1.24±0.17 g and 1.82±0.29 g, respectively, which were much lower than that of the wild-type strain (7.76±1.96 g). Furthermore, the sclerotia produced by *SSITL* silenced transformants did not germinate carpogenically.

The virulence of *SSITL* silenced transformants were significantly reduced and only small lesions were developed on leaves of *Brassica napus* (Figure 3F). The lesions induced by A10 and B6 were about 1.5 cm and 1.7 cm in diameter, respectively, while the lesion induced by the wild-type strain was about 3.5 cm in diameter. Smaller lesions on other hosts, such as *A. thaliana*, lettuce, cucumber and soybean, were also observed when A10 and B6 were inoculated (data not shown).

Taken together, *SSITL* has pleiotropic effects on virulence, hyphal growth, sclerotial development and germination.

### SSITL is Secreted to Cell Walls of *S. sclerotiorum* and the Extracellular Matrix

**Figure 4:**
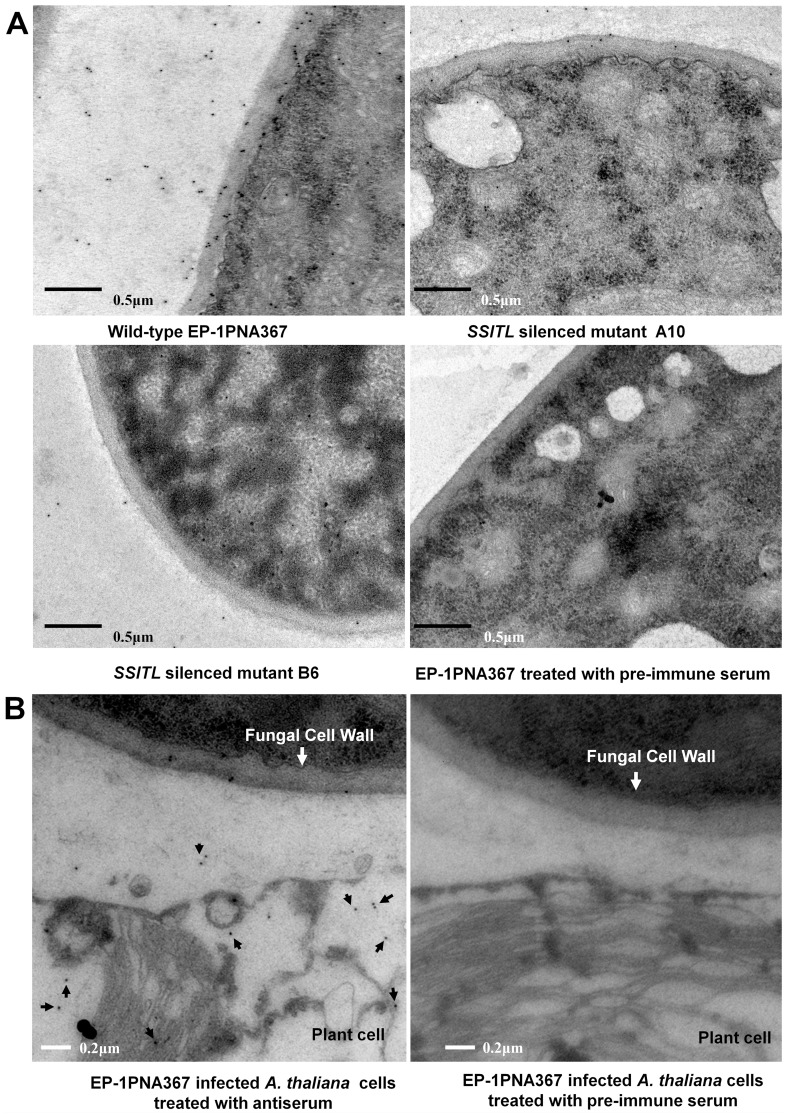
Immunolocalization of SSITL of *S. sclerotiorum* during hyphal growth and infection. (A) Hyphae for ultrathin sections were collected from 3-day-old colony grown on PDA at 20°C. Ep-1PNA367 and the *SSITL* silenced transformants A10 and B6 were incubated with the antiserum raised by immunizing rabbits with SSITL, respectively; the hyphae of Ep-1PNA367 which was treated with the pre-immune serum were used as control. (B) Immunolocalization of SSITL (the arrow point) in *A. thaliana* leaf cells infected by Ep-1PNA367 at 12 hpi. Left: Treated with antiserum; Right: Control sections treated with pre-immune serum. Hyphal agar discs were cut from colony margins and inoculated to the leaves of Arabidopsis for 12 hr before the lesion margin was collected for ultrathin sectioning analysis.

**Figure 5:**
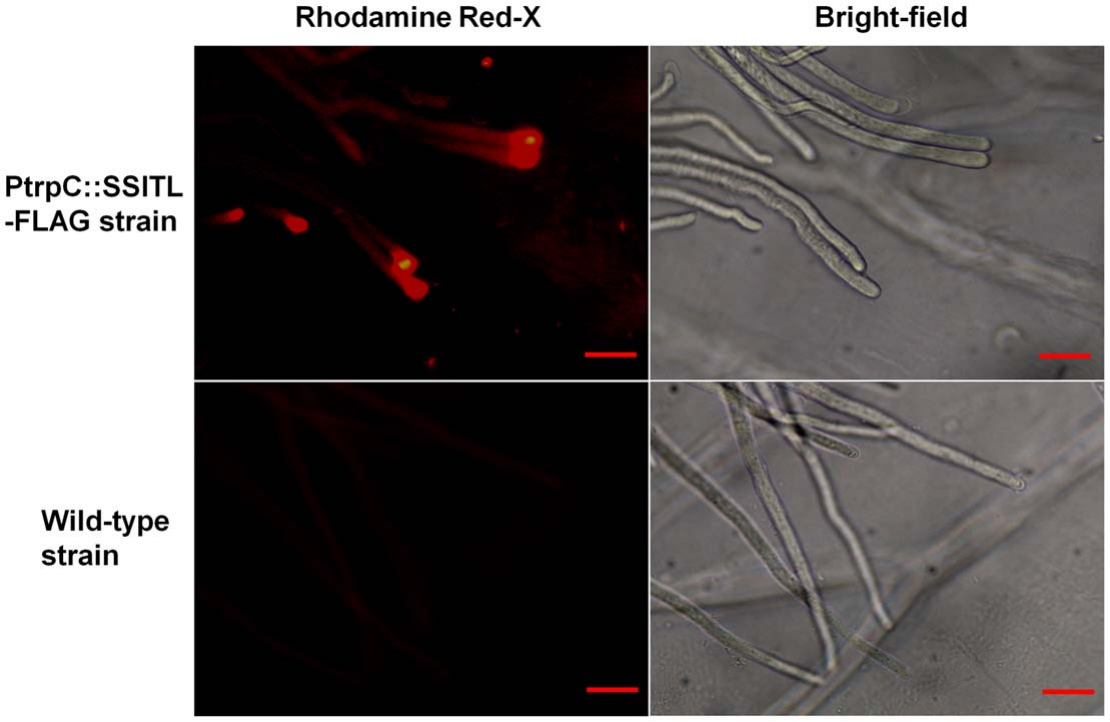
Immunofluorescence detection of SSITL during *S. sclerotiorum* infecting on onion bulb epidermis. A transgenic strain of *S. sclerotiorum* in which an SSITL:Flag tag fusion protein was expressed using *PtrpC.* Onion bulb epidermis was inoculated with strains for 12–24 hr at 20°C, and was used for immunofluorescence observations under a Nikon Eclipse 80i fluorescent microscope (Nikon, Japan).

The subcellular localization of the SSITL in hyphae was detected by immunogold labeling. Our results indicated that SSITL located mainly on the fungal cell wall, and was also observed in the extracellular matrix and cytoplasm (Figure 4A). The accumulation of SSITL on hyphal cell walls of A10 was significantly lower than the wild-type strain (Figure 4A) in accordance with *SSITL* being silenced. No gold labeled particles were observed on the cell walls of the wild-type or mutant strains treated with pre-immune serum (Figure 4A). Immunofluorescence studies also showed that the SSITL:Flag fusion protein was secreted through the hyphal tip during infection of onion epidermis (Figure 5), which is consistent with other reports that the secretory proteins were observed accumulating at the tips of hyphae during infection of *Magnaporthe oryzae* and other fungi [48]–[50]. Furthermore, when *S. sclerotiorum* infected Arabidopsis, gold labeled particles were detected in the plant cells (Figure 4B), while control sections displayed no labeling signals in fungal or plant cells (Figure 4B). Thus, the SSITL was secreted to the host cell and may have played an important role in promoting the infection.

### *SSITL* Silenced Transformants Induce both Local and Systemic Resistance in Arabidopsis

**Figure 6:**
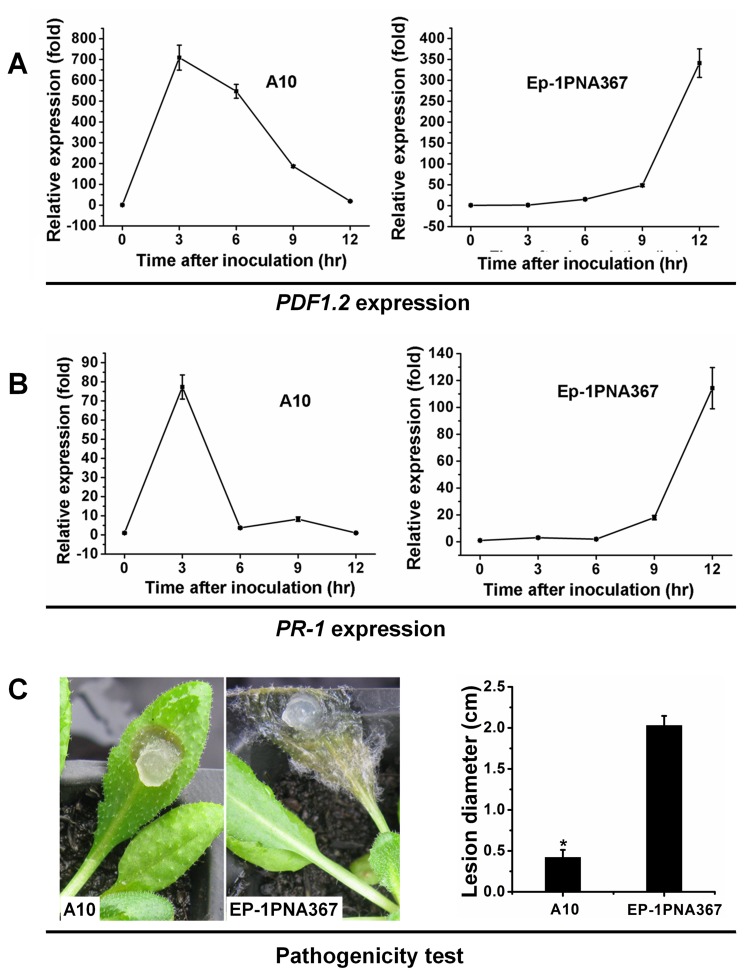
Expression of *PDF1.2* and *PR-1* induced by transformants of *S. sclerotiorum* at locally inoculated leaves of *A. thaliana* at the early stage of infection. The expression of *PDF1.2* (A) and *PR-1* (B) on leaves inoculated with silenced transformant A10 or with the wild-type strain Ep-1PNA367. The relative levels of transcript were calculated by the comparative Ct method. Expression on leaves of *A. thaliana* inoculated with pathogen for 0 hr was set as one. Transcript levels of *GAPDH* of Arabidopsis were used to normalize different samples. Bars represent means and standard deviations (three replications). (C) Lesions induced by transformant A10 and the wild-type strain Ep-1PNA367 on leaves observed at 20°C for 36 hr. Asterisks indicate statistical differences between the lesions diameter induced by A10 and Ep-1PNA367 (*P*<0.05).

*SSITL* silenced transformants, like A10, produces a considerable amount of oxalic acid, but are still attenuated in virulence on hosts. We suspected that infection by transformants might trigger resistance in the host. To experimentally investigate this possibility transcript accumulation of pathogenesis-related genes *PR-1* and plant defensin *PDF1.2* in *A. thaliana* were examined. When leaves were inoculated with *SSITL* silenced transformants A10, rapid and increased levels of transcriptions of these two genes were observed at the early stages of infection. The expression of *PDF1.2* and *PR-1* on inoculated leaves increased 708 fold and 77 fold at 3 hpi respectively, as compared with those at 0 hpi (Figure 6A and B). High expression of *PDF 1.2* was still observed at 9 hpi, and then began to drop, but the transcripts levels of *PDF 1.2* at 12 hpi were still higher than at 0 hpi (Figure 6A). However, the expression of *PR-1* dropped quickly and the expression level of *PR-1* at 6 hpi was very close to that of 0 hpi (Figure 6B). The results were similar to the previous report that much higher expression levels of defense response genes were induced at early stage in tomato leaves when inoculated with attenuated virulent *B. cinerea* isolate compared with that of virulent isolate [3]. In contrast, when leaves were inoculated with the wild-type strain Ep-1PNA367, the transcript accumulations of *PDF1.2* and *PR-1* were low at the early stages, and then they increased gradually and reached a peak at 12 hpi (Figure 6A and B); similar resistance reactions of Arabidopsis to virulent *S. sclerotiorum* and *B. cinerea* strains have also been detected previously [15], [16], [51].

With respect to virulence, lesions on Arabidopsis induced by A10 were much smaller in diameter than that of the wild-type strain Ep-1PNA367 (Figure 6C), consistent with the higher expression levels of these defense responses genes in the Arabidopsis inoculated with A10 compared with that of the wild-type. SSITL therefore may be required to suppress host defense responses.

**Figure 7:**
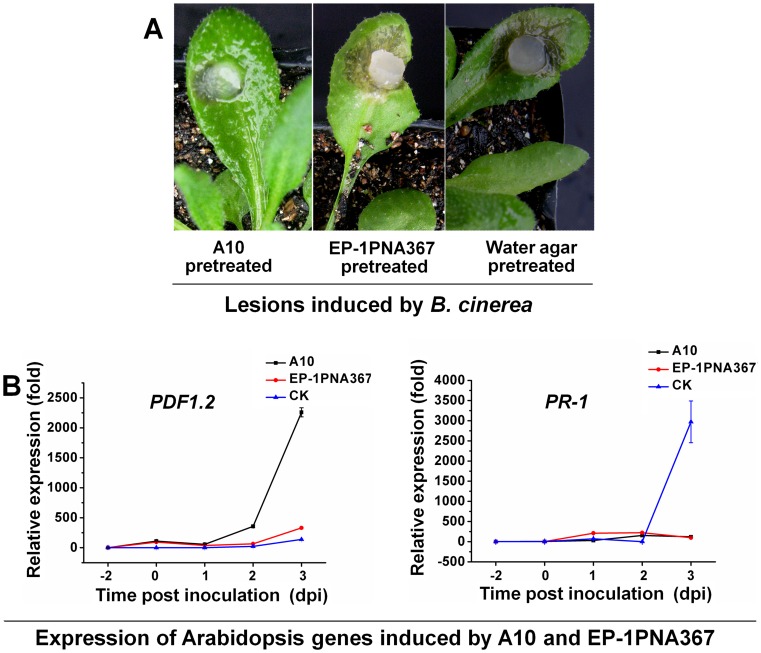
Strong systemic resistance induced by *SSITL* silenced transformants of *S. sclerotiorum*. (A) The lesions induced by *B. cinerea* with the lower leaves being pretreated with *SSITL* silence transformant A10, Ep-1PNA367 and the water agar plugs (CK), respectively. Leaves were inoculated with A10 or the wild-type *S. sclerotiorum* or water agar for two days before inoculated leaves were cut and then inoculated with *B. cinerea* at 20°C for 72 h. (B) Expression of *PDF1.2* on upper leaves of inoculated plants pretreated with A10. Expression in un-inoculated leaves of *A. thaliana* was set as level 1. At 2 dpi, leaves of Arabidopsis were inoculated with A10 or the wild-type strain *S. sclerotiorum* or water agar and, at 0 dpi, and upper healthy leaves were inoculated with *B. cinerea*. Expression of *GAPDH* was used to normalize. Bars represent means and standard deviations (three replications).

To examine whether systemic resistance occurs, mycelial plugs of the wild-type strain and A10 were inoculated on the leaves of Arabidopsis. At 48 hpi, upper leaves of the same plant were inoculated with *B. cinerea* for a further 72 hr. The results showed that when pre-treated with A10, the secondary infection lesions caused by *B. cinerea* were significantly smaller than those pre-treated with the wild-type strain and water agar plugs (control) (Figure 7A).

Real-time RT-PCR amplification results showed that expression of *PDF1.2* on the upper leaves of plant pre-treated with A10 was much higher than those plants pre-treated with the wild-type strain of *S. sclerotiorum* or water agar. The induced expression of the *PDF1.2* corresponds with the disease severity but not the *PR-1* (Figure 7A and B).

These results showed that A10 can induce both local and systemic resistance in *A. thaliana*, while the wild-type cannot induce systemic resistance or possibly suppress this resistance, and suggested that both JA/ET-dependent and SA-dependent signal pathways are involved in local resistance of Arabidopsis to *S. sclerotiorum* infection, but JA/ET-dependent signal pathway plays a more important role in both the local and the systemic resistant reactions. However, both local and systemic resistances are suppressed by the wild-type strain of this pathogen at the early stage of infection.

**Figure 8:**
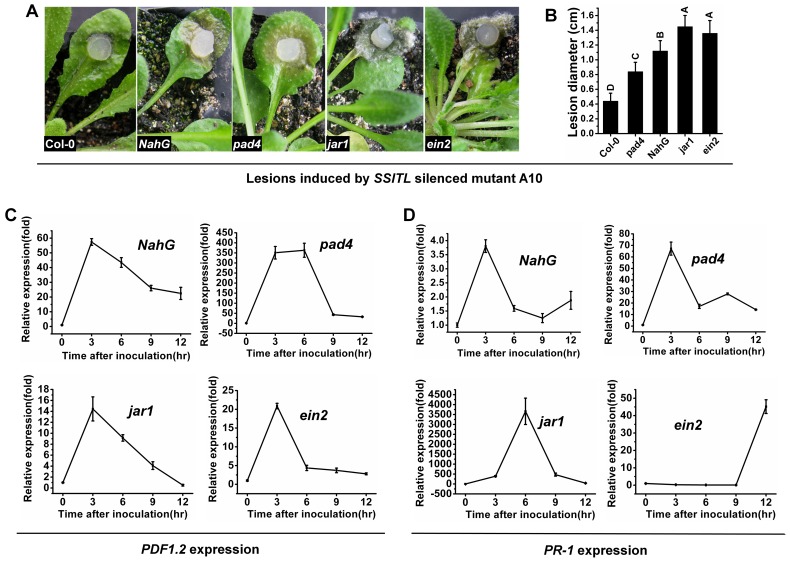
Enhanced susceptibility to A10 produced by disruption of JA/ET and SA signal pathway of Arabidopsis. (A, B) A10 induced larger lesions on the leaves of Arabidopsis mutant *jar 1* and *ein2*, and mutant *pad 4* and transgenic line *NahG* were more susceptible than the wild-type of *A. thaliana*. Different letters in the graph indicate statistical differences, *P* = 0.01. (C, D) The relative expression of *PDF1.2* and *PR-1* gene in Arabidopsis mutants and transgenic line *NahG* inoculated with A10. Plants were incubated at 20°C for 36 hr after being inoculated with active mycelial agar discs of A10. Bars represent means and standard deviations (three replications).

**Figure 9:**
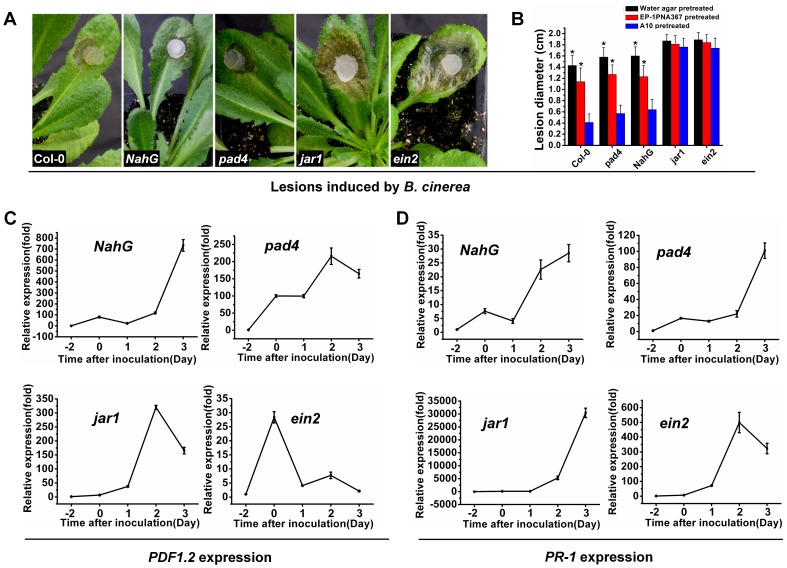
Failure of A10 to induce systemic resistance in mutants of Arabidopsis disrupted in the JA/ET signal pathway. (A) The lesions caused by *B. cinerea* on *A. thaliana* mutations and transgenic line *NahG* for 72 hr at 20°C after being pretreated with A10. (B) The lesions caused by *B. cinerea* on *A. thaliana* for 72 hr at 20°C after being pretreated with A10 (blue), Ep-1PNA367 (red) and water agar plugs (black). Asterisks indicate statistical differences from the A10 pretreated (*P*<0.05). (C, D) The expression of *PDF1.2* and *PR-1* during the infection of *B. cinerea* on *A. thaliana* mutations and transgenic line *NahG* after being pretreated with A10. Bars represent means and standard deviations (three replications).

To further validate these conclusions, *A. thaliana* mutant *pad4, jar1, ein2* and *NahG* Arabidopsis were inoculated with A10. The lesions induced by A10 on *jar1* and *ein2* were obviously larger than lesions on *pad4* and *NahG*, and the latter were also larger than those on the wild-type Arabidopsis (Figure 8A and B), which correlates with the lower transcript accumulations of the defense responses genes (Figure 8C and D). A10 was also inoculated for 48 hr on the leaves of *pad4, jar1, ein2* and *NahG*, respectively, and *B. cinerea* was inoculated on the upper leaves after pre-inoculated leaves were removed as described above. The lesions induced by *B. cinerea* on the leaves of *jar1* and *ein2* were obviously larger than those on leaves of *pad4* and *NahG*, and than those on the wild-type Arabidopsis as well (Figure 9A and B), which is consistent with the lower transcript accumulation of *PDF1.2* (Figure 9C and D).

### Transient and Constitutive Expression of *SSITL* in Host Plants Leads to be more Susceptible to *S. sclerotiorum*

**Figure 10:**
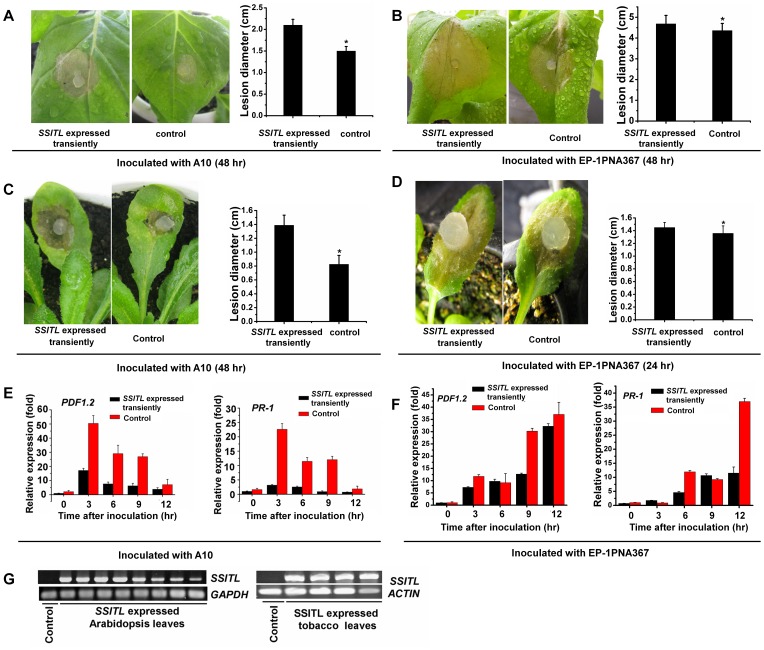
Enhanced susceptibility to *SSITL* silenced transformant A10 induced by the transient expression of *SSITL* in the host plants. (A, B) The lesions induced by silenced transformant A10 and the wild-type strain Ep-1PNA367 on the leaves of tobacco (*Nicotiana benthamiana*) for 48 hr at 20°C. (C, D) The lesions induced by silenced transformant A10 and the wild-type strain Ep-1PNA367 on the leaves of *A. thaliana* Col-0 for 48 hr and 24 hr at 20°C, respectively. *SSITL* was expressed transiently in plants leaves by infiltrating with Agrobacterium GV3101 strain carrying *SSITL* expression vector. The leaves infiltrated with GV3101 carrying empty vector were selected as control (CK). Asterisks indicate statistical differences from the control (*P*<0.05). (E, F) Transcript levels of *PDF1.2* and *PR-1* in the *SSITL* transiently expressed *A. thaliana* leaves (black) after being respectively inoculated with silenced transformant A10 and the wild-type strain Ep-1PNA367 for 3, 6, 9 and 12 hr, with the plant leaves infiltrated with the GV3101 carrying empty vector being sampled for control (red). (G) Analysis the *SSITL* expression in the transiently expressed leaves of *A. thaliana* and tobacco (*N. benthamiana*) with RT-PCR. The leaves infiltrated with the GV3101 carrying empty vector were selected as control (CK). The *A. thaliana GAPDH* and *N. benthamiana actin* genes (see primers in Table 1) were used to normalize different samples.

**Figure 11:**
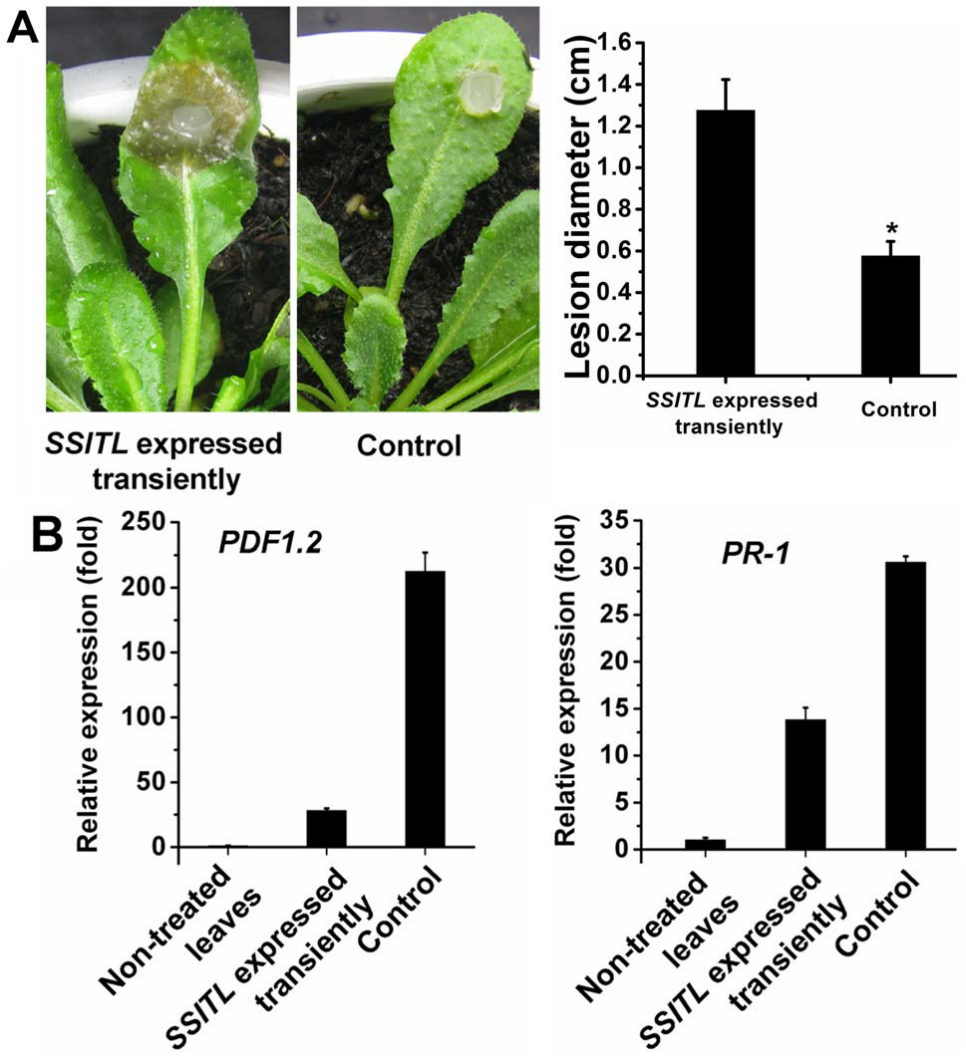
Suppression of the systemic resistance induced by *SSITL* silenced transformant A10 in the Arabidopsis plants of *SSITL* transient expression. (A) The lesions induced by *B. cinerea* on upper leaves of plants after the *SSITL* transiently expressed leaves were inoculated with A10 for 48 hr. The lesions were induced by *B. cinerea* at 20°C and were measured at 72 hpi. Asterisks indicate statistical differences from the control (*P*<0.05). (B) The relative transcript levels of *PDF1.2* and *PR-1* in the upper leaves of plant after the *SSITL* transiently expressed leaves were inoculated with A10 for 48 hr. Plants infiltrated with GV3101 carrying empty vector only, served as control.

To further verify that SSITL impacts the defense responses of host plants, transient and constitutive expression of *SSITL* in host plants was conducted. On *SSITL* transiently expressed leaves of tobacco (*Nicotiana benthamiana*) and *A. thaliana* (Figure 10G), lesions induced by A10 were significantly larger than those on control leaves (Figure 10A and C). Following inoculation with A10, the relative expressions of defense genes *PDF1.2* and *PR-1* were also much lower than in control leaves (Figure 10E). Furthermore, on *SSITL* transiently expressed leaves, the lesions induced by Ep-1PNA367 were still slightly larger than those on control leaves (Figure 10B and D); and when inoculated with Ep-1PNA367, the expression of defense genes *PDF1.2* in *SSITL* transiently expressed leaves was slightly suppressed (Figure 10F), but the suppression was not so obvious as in A10 inoculated leaves. Systemic resistance induced by A10 was also suppressed. Compared with control, when A10 was inoculated on *SSITL* transiently expressed leaves for 48 hr, larger lesions on the upper leaves of Arabidopsis plant were induced by *B. cinerea* (Figure 11A), which is consistent with the reduced transcript levels of defense genes in the upper leaves (Figure 11B).

**Figure 12:**
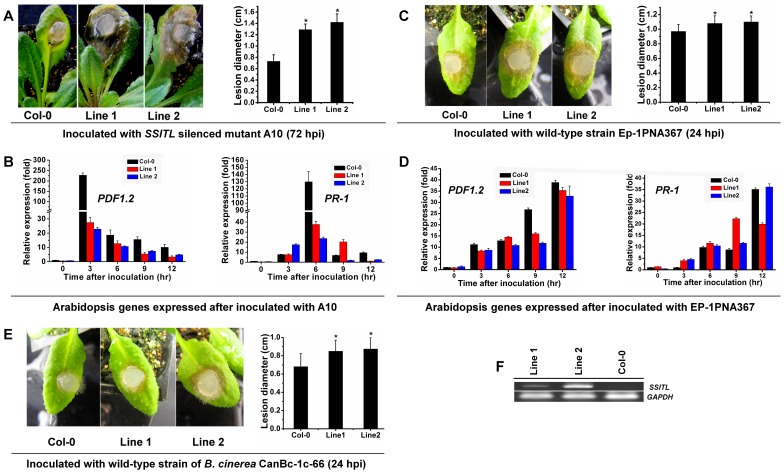
Suppression of resistance induced by A10 in *SSITL* transgenic lines of *A. thaliana*. (A) Lesions on the leaves of *SSITL* transgenic lines (line 1 and line 2) and the wild-type *A. thaliana* Col-0 induced by A10. Lesions were measured at 72 hpi. Asterisks indicate statistical differences from the wild-type Col-0 (*P*<0.05). (B) *PDF1.2* and *PR-1* expression in the wild-type Col-0 (black), *SSITL* transgenic line 1 (red) and line 2 (blue) after inoculated with A10. (C) Lesions induced by Ep-1PNA367 on the wild-type *A. thaliana* Col-0, *SSITL* transgenic line 1 and line 2 at 20°C for 24 hr. Asterisks indicate statistical differences from the wild-type Col-0 (*P*<0.05). (D) *PDF1.2* and *PR-1* expression in the wild-type Col-0 (black), *SSITL* transgenic line 1 (red) and line 2 (blue) after inoculated with Ep-1PNA367. (E) Lesions induced by *B. cinerea* on the wild-type *A. thaliana* Col-0, *SSITL* transgenic line 1 and line 2 at 20°C for 24 hr. Asterisks indicate statistical differences from the wild-type Col-0 (*P*<0.05). (F) The analysis of *SSITL* expression in transgenic lines (line 1 and line 2) of *A. thaliana* with RT-PCR. *A. thaliana GAPDH* (see primers in Table 1) was used to normalize different samples.

*SSITL* was transformed successfully into Arabidopsis and was constitutively expressed in transgenic lines (Figure 12F). The transgenic lines were inoculated with A10 and Ep-1PNA367, respectively. Results showed that the lesions on the leaves of transgenic lines induced by A10 were obviously larger than those on the wild-type Col-0 (Figure 12A), and the relative expressions of *PDF1.2* in the leaves of transgenic lines were significantly lower than that in the wild-type Col-0 after being inoculated with A10 (Figure 12B). Furthermore, the lesions on the leaves of transgenic lines induced by the wild-type strain Ep-1PNA367 were still slightly larger than those on the wild-type Col-0 (*P*<0.05) (Figure 12C) in spite of the relative expression of defense genes *PDF1.2* between leaves of *SSITL* transgenic lines and the wild-type were not significantly different after being inoculated with Ep-1PNA367 (Figure 12D). And the lesions on the leaves of transgenic lines induced by the wild-type of *B. cinerea* were also slightly larger than those on the wild-type Col-0 at 24 hpi (*P*<0.05) (Figure 12E). Thus, these data are consistent with the ability of SSITL to suppress JA/ET signal pathway mediated resistance in host plants at very early stage of infection and make hosts more susceptible to *S. sclerotiorum*.

## Discussion

In this paper, we investigated an integrin-like gene (*SSITL*) of *S. sclerotiorum.* This gene was significantly downregulated in the presence of hypovirulence associated mycovirus SsDRV. SSITL is an extracellular protein involved in virulence of *S. sclerotiorum*. Targeting silencing of *SSITL* in *S. sclerotiorum* resulted in the reduction of virulence, reduced hyphal polarity, and decreased sclerotia both qualitatively (morphology/size) and quantitatively (numbers). Sclerotia produced by *SSITL* silenced transformants were also defective in carpogenic germination. Moreover, we found that SSITL is likely to be an effector and is involved in suppression of host resistance mediated by JA/ET signal pathway at an early infection stage.

When inoculated with the *SSITL* silenced transformant, strong and quick defense response of Arabidopsis to *S. sclerotiorum* was induced at the very early stage of infection (about 3 hpi) based on the highly expression of *PDF1.2* and *PR-1*, suggesting that Arabidopsis quickly responds to initiate resistance against *SSITL* silenced transformant. Both *PDF1.2* and *PR-1* were highly induced at 3 hpi and the high level expression of *PDF1.2* lasted to 9 hpi, while the expression of *PR-1* dropped quickly at 6 hpi (Figure 6A and B). The high expression of *PDF1.2* suggested that JA/ET signal pathway is involved in counteracting the infection of *S. sclerotiorum*. And also, *SSITL* silenced transformant A10 can induce larger lesions on Arabidopsis mutant *jar1* and *ein2* than on mutant *pad4* and *NahG* (Figure 8A and B), further confirmed the important contribution of JA/ET signal pathway for Arabidopsis against *S. sclerotiorum*. Our finding is consistent with other reports for necrotrophic pathogens [3], [12]–[16], [51].

While inoculating Arabidopsis with the wild-type strain of *S. sclerotiorum*, the expressions of *PDF1.2* and *PR-1* in inoculated leaves were suppressed at the early stage of infection, and were only detectable after 9 hpi in our experiment (Figure 6A and B), similar results were also observed in other studies [15], [51]. This defense response is too late to counteract necrotrophic pathogen, such as *S. sclerotiorum*, since pathogens have already colonized on their host. Small lesions at the inoculation sites can be observed under microscopy at 6 hpi; furthermore, typical and macroscopic necrosis lesions induced by *S. sclerotiorum* around the inoculation sites can be observed easily with naked eyes at about 12 hpi. In this paper, we found that the wild-type strain of *S. sclerotiorum* can suppress the defense response of Arabidopsis at the early stage of infection to facilitate its infection while the *SSITL* silenced transformant failed or postponed to suppress the defense response, thus, SSITL is involved in the suppression of host defense at the early stage of infection.

Usually, the strong defense against the wild-type strain of *S. sclerotiorum* at the early stage of infection is not detectable, which means that the defense is most likely to be suppressed or postponed by this pathogen. If suppression is a means by which *S. sclerotiorum* is successful as a pathogen, then it is not surprising that *S. sclerotiorum* may secrete pathogenicity factors to aid in the suppression of host resistance. Previous studies on the pathogenicity of necrotrophic pathogens mainly focus on toxins (including proteinaceous effectors), plant cell degrading enzymes and proteinases [20], [22], [52]–[56]. Oxalic acid is considered a key pathogenicity factor for the killing of host cells and tissues by *S. sclerotiorum* [57], and it is also involved in suppressing host resistance and interrupting the host physiology rather than as a direct killer [11], [21], [22], [58]. However, this topic is also one of increasing complexity; several mutants of *S. sclerotiorum* produce a considerable amounts of oxalic acid, but do not infect the plant; the *SSITL* transformant and the virus mediated hypovirulent strain Ep-1PN, also produce significant amounts of oxalic acid, but virulence is weak [59]; in addition, the mutant cannot produce oxalic acid, but can still infect plant [60]. Recently, Williams *et al* [11] found that reactive oxygen species (hydrogen peroxide) was virtually absent in DAB stained leaf inoculated with the wild-type strain of *S. sclerotiorum*, while leaves inoculated with an oxalic acid deficient mutant A2 displayed strong DAB staining surrounding the infection point, and they believed that oxalic acid suppresses host defenses by manipulating the host redox environment at 8 hpi, an early stage of infection. Our experimental results also suggested that the resistance of host may occur at a very early stage, even earlier than 3 hpi, and SSITL is involved in the suppression of the JA/ET signal pathway mediated resistance.

Bioinformatics analyses indicate that SSITL is likely a protein similar to the integrin-like protein α-subunit of animals. The majority of studies with integrins have focused on mammalian systems, while rarely on phytopathogenic fungi. Particularly noteworthy is the fact that integrins can signal through the cell membranes in either direction [25]–[27]. Thus the presence of SSITL may have functional relevance and maybe attributed to several pathways. SSILT is a secretory protein and the distinct difference between SSITL and typical integrins is the absence of a transmembrane domain in SSITL. Thus, SSITL may be mobile and is not directly anchored on membranes. Our finding that SSITL was secreted into host cells during infection of onion epidermis and was also detected in the leaf cells of Arabidopsis, is consistent with the mobile characteristic of SSITL. It will be important to determine binding partners for SSITL since this protein may interact with a host receptor and/or a fungal protein.

The mechanism by which SSIT suppresses defense of Arabidopsis against *S. sclerotiorum* infection is unknown. Previously, *C. albicans* was found to express surface proteins with functional and antigenic characteristics of human complement receptors type 3 (CR3), a member of the integrin superfamily, suggesting that *C. albicans* was using this form of molecular mimicry to elude phagocytosis [61]. In rice, the LysM domain–containing pattern recognition receptor protein CEBiP, recognizes and directly binds chitin oligosaccharides released from the cell walls of fungal pathogens may induce chitin-triggered immune responses in rice cells [62], [63]. Recent studies demonstrated that the secretory effector protein Slp1 of *Magnaporthe oryzae* also contains the LysM domains as observed in CEBiP, and Slp1 competes with CEBiP for binding to chitin oligosaccharides, thus preventing chitin-triggered immunity in rice [48]. As mentioned above, plants also have integrin-like proteins, and NDR1, a pathogen-induced protein required for Arabidopsis disease resistance [64], [65], was identified as an integrin-like protein [30]. Further studies showed that NDR1 interacts with RIN4 initiating a resistant response [66]. Interestingly, we have also found that both the *A. thaliana* NDR1 and *S. sclerotiorum* SSITL protein possess the RGD-like motif NGD (Asn-Gly-Asp), and the NGD motif in *A. thaliana* NDR1 is involved in defense signaling following pathogens infection [30]. It is possible that *S. sclerotiorum* uses SSITL to mimic NDR1 or other plant integrin-like proteins as receptor to hold pathogenicity factors, and then suppresses and/or interfere with the host resistance.

The importance of integrin-like proteins in other fungi or fungi-like organisms also was illuminated. In *U. appendiculatus*, integrin-like proteins are involved in the transmission of physical signals from the leaf surface to initiate the formation of appressoria [32]. The *int1* gene of *C. albicans* contributes to polar filamentous growth and induces the growth of highly polarized buds [67]. In the fungus-like organism, *Saprolegnia ferax*, an integrin protein, mediates cytoplasm-wall adhesion and affects the growth rates of tip hyphae [67]. Besides facilitating pathogenesis by suppressing the host defense, the SSITL is also involved with proper hyphal growth and sclerotial development. *SSITL* silenced transformants showed physiological debilitating phenotypes, including slow growth, excessive tip branching, frequent abortion of sclerotial development and production of small sclerotia that fail to germinate carpogenically. We have also tried several times to delete *SSITL* gene in *S. sclerotiorum*, but failed, considering the importance mentioned above, *SSITL* gene is most likely to be an essential gene for *S. sclerotiorum* to survive and deletion of this gene will be fatal for *S. sclerotiorum*. Surprisingly, only a few homologs of SSITL were found in other fungi, such as *Aspergillus* spp., *B. cinerea*, *Talaromyces stipitatus* and *Fusarium oxysporum*, and the functions of SSITL homologs in these fungi are yet to be explored.

In summary, we have identified a gene encoding a secretory integrin-like protein (SSITL) from *S. sclerotiorum.* SSITL is involved in suppressing host defense at early stages of infection. This finding enhances our understanding on pathogenicity of *S. sclerotiorum* beyond necrotrophic stage. However, our finding also arises more questions to be answered, such as, what kind of signal promotes *SSITL* expression during the early stage of infection? Does this signal come from fungus innately or from host? And how does SSITL suppress host resistance defense?
